# Aroma Profiling and Sensory Association of Six Raspberry Cultivars Using HS-SPME/GC-MS and OPLS-HDA

**DOI:** 10.3390/foods14213599

**Published:** 2025-10-22

**Authors:** Jovana Ljujić, Boban Anđelković, Ivana Sofrenić, Katarina Simić, Ljubodrag Vujisić, Nevena Batić, Stefan Ivanović, Dejan Gođevac

**Affiliations:** 1 Faculty of Chemistry, University of Belgrade, Studentski trg 12–16, 11000 Belgrade, Serbia; jovanalj@chem.bg.ac.rs (J.L.); ivanasofrenic@chem.bg.ac.rs (I.S.); ljubaw@chem.bg.ac.rs (L.V.); 2 Institute of Chemistry, Technology and Metallurgy, National Institute of the Republic of Serbia, University of Belgrade, Njegoševa 12, 11000 Belgrade, Serbia; katarina.simic@ihtm.bg.ac.rs (K.S.); stefan.ivanovic@ihtm.bg.ac.rs (S.I.); dejan.godjevac@ihtm.bg.ac.rs (D.G.); 3 Faculty of Agriculture, University of Belgrade, Nemanjina 6, Zemun, 11080 Belgrade, Serbia; nevena.momirovic@agrif.bg.ac.rs

**Keywords:** raspberry aroma, sensory analysis, headspace solid-phase micro extraction, GC-MS, OPLS-HDA

## Abstract

In this study, six club raspberry varieties were examined for their aromatic profiles and sensory qualities, and statistical approaches were used to determine how aroma components affect consumer impressions. Analysis of the aroma’s chemical composition was performed utilizing headspace SPME and GC-MS. MS-DIAL -v5.5.250627 software was used to identify components from commercial libraries, after 10 repetitions for each variety, followed by manual verification. A sensory evaluation of fresh fruits, with 55 volunteers, was statistically analyzed and linked to chemical composition using multivariate analysis and the OPLS-HDA classification method, which was employed for the first time. Tula Magic was scored the highest in the sensory evaluation compared to Adelita, Himbo Top, Glen Dee, San Rafael, and Cascade Harvest. 2-Heptanol (fresh, lemongrass-like, herbal, floral, fruity, green), heptanal (fresh, aldehydic, fatty, green, herbal), and 2-methyl-6-hepten-1-ol (oily-green, herbaceous-citrusy) separated Tula Magic from the other varieties assessed. The same components were recognized in OPLS as positive contributors to the flavor score, while terpenoids like trans-β-ionone, α-ionone, and α,β-dihydro-β-ionone, as well as 2-heptanone, scored slightly lower. This suggests that a fine balance between the individual components is key to the overall aroma sensation.

## 1. Introduction

Due to their unique and authentic taste, as well as the wealth of bioactive compounds with significant health benefits contained in them, the consumption of raspberries has seen a constant increase over the last few decades. Modern trends in raspberry cultivation increasingly focus on improving the nutritional composition and organoleptic properties of the fruit, unlike earlier approaches that prioritized physical characteristics [1]. Numerous scientific studies emphasize the role of bioactive components of raspberry in the prevention of various chronic diseases, including diabetes, cardiovascular diseases, malignant diseases, and neurodegenerative disorders [2]. Worldwide, 673,720 t of raspberry is produced per year. After the Russian Federation, with 98,700 tones production per year, Serbia is the second largest producer of raspberries in the EU and the third largest in the world. The war in Ukraine and the embargo on the export of agricultural products from Russia put Serbia in a privileged position, and despite the decrease in demand and purchasing power throughout the EU, the demand for Serbian raspberries has increased, and so has its price. Since the 1970s, Willamette Polka and Polana were traditionally the dominant variety grown in Serbia, accounting for about 90% of the area and plantings. The majority of these raspberries (over 90%) are exported as frozen [3]. To increase revenue and expand into the fresh produce market, a pilot plantation with six club varieties was established on the Zeleni Hit experimental field to test their potential and adaptability to the Serbian microclimate.

Taste and aroma represent basic sensory characteristics that significantly determine the quality and consumer acceptability of food products [4]. Raspberry aroma is a complex mixture of volatile compounds that are formed as a product of various biochemical processes, including enzymatic reactions, microbiological fermentation, and chemical transformations during processing and storage [5]. These compounds play a key role in the formation of the characteristic and recognizable aromatic profile of raspberries, which is important not only for the perception of the sensory quality of the fruit but also for its resistance to mold [6]. Depending on the variety, the profile of volatile compounds can differ, both qualitatively and quantitatively [7]. Various biotic and abiotic factors, present throughout the entire plant production, influence the variety and concentration of these compounds [4]. During ripening, the raspberry fruit goes through different stages, during which its volatile profile changes. The level of certain volatile compounds increases with maturation, while in others it may remain constant or even decrease after reaching full maturity [8]

Volatile compounds are usually small organic molecules with a molecular weight less than 300 Daltons. So far, almost 300 volatile compounds from raspberry fruits have been characterized, including terpenes, esters, alcohols, isoprenoids, aldehydes, acids, and ketones [7]. Raspberry ketone, α- and β-ionone, linalool, geraniol, nerol, (Z)-3-hexenol, α-terpineol, β-ocimene, β-pinene, β-damascenone, furaneol, (E)-2-hexenal, heptanal, hexanal, 1-octanol, ethyl 2-methylpropanoate, and benzaldehyde contribute to the raspberry aroma [8]. However, only a few of them represent the key bearers of the raspberry aroma [9]. Raspberry ketone provides the typical sweet aroma, while α- and β-ionone contribute to pronounced floral notes. These volatile compounds represent the key carriers of the raspberry aroma, regardless of the genotype of the variety. Furthermore, monoterpene alcohols, geraniol and linalool, contribute significantly to the floral and fruity notes of the raspberry’s aromatic profile [10]. Apart from them, typical volatile compounds of raspberry are also aldehydes, namely decanal, hexanal, and nonanal [11].

Aroma extraction and quantification is a historically long process. Liquid–liquid extraction was one of the first applied methods, with the fact that the efficiency of the extraction depended to a significant extent on the choice of adequate solvent [8]. However, techniques based on the use of solvents may have certain disadvantages, such as the risk of sample contamination, analyte loss during the concentration process, or environmental problems due to the large consumption of organic solvents [12]. After a while a more advanced method developed by Nickerson and Lickens, known as simultaneous distillation extraction (SDE), was introduced. Although this technique enabled the efficient extraction of aromas, it proved to be time-consuming and required a relatively large amount of sample, leading to its limited use in modern laboratories [5].

To overcome long-term extraction processes and reduce the consumption of resources, more sustainable and environmentally acceptable extraction techniques are being used in modern analytical methods for aroma testing. Among them, the technique of solid-phase microextraction (SPME) stands out in particular [13]. This technique enables the simultaneous extraction and concentration of volatile compounds using a fiber, which can be coated with different adsorbents. Aroma extraction can be performed by directly immersing the fiber in a liquid sample (Direct Immersion, DI-SPME) or by exposing the fiber to the gas phase in the space above the sample (Head Space, HS-SPME) [14]. The type and thickness of the fiber coating are the most important characteristics that determine the analytical performance of HS-SPME. The selectivity of the extraction is affected by the type of fiber used. Similar to conventional gas chromatography (GC) stationary phases, non-polar fibers are used for non-polar analytes and polar for polar ones [10].

Given that the demand for raspberries is constantly growing at the global level throughout the year, it is necessary to introduce new varieties with higher nutritional and commercial quality, which will also be adapted to the agro-ecological conditions of the area. Consequently, the aromatic profile and sensory characterization have a significant impact on the consumer acceptability of the fruit. In our work, the volatile profiles of the club varieties Tula Magic, Glen Dee, San Rafael, Adelita, Cascade Harvest, and Himbo Top, which were grown on the sample field of the Zeleni hit company, were examined. The Swiss-bred ‘TulaMagic’ raspberry, a hybrid between ‘Autumn Bliss’ and ‘Tulameen’, bears in summer [15]. Sweet, fragrant fruit and root rot resistance are its hallmarks. The summer-bearing raspberry Glen Dee has late-season fruiting; large, firm, conical berries; and spine-free, erect canes that are easy to handle [16]. The Jackman Humanities Institute at the University of Toronto in Inverurie, Canada, developed this variety, which professional growers like for its high yields, shelf life, and picking ease. Adelita, introduced by Planasa in 2012, is a single raspberry variety notable for its winter productivity [17]. It grows huge, tasty, firm, bright red berries and is easy to manage and market in Spain, Portugal, Morocco, and Mexico. The shrub can be planted in autumn, spring, and winter for a steady fruit supply. WSU, OSU, and the USDA released the Cascade Harvest raspberry in 2014 as a single red raspberry cultivar. It could replace the Pacific Northwest’s ‘Meeker’ cultivar because of its large, firm fruit, disease tolerance (particularly root rot and Raspberry Bushy Dwarf Virus), thornless canes, and machine harvestability [16]. ‘Himbo Top’ is not a classification of raspberries, but rather a distinct type. The registered trademark is Himbo-Top^®^, and the variety is also referred to as ‘Rafzaqu’. launched in 2008, Himbo Top is a renowned and prolific primocane (fall-bearing) red raspberry distinguished by its substantial, vibrant red fruit and exceptional vigor [18]. ‘San Rafael’ is a type of deciduous shrub that exhibits growth. The variety is categorized as floricane, producing fruit on second-year wood. Recent harvests have surpassed 500 g per plant, indicating a production enhancement of over 20% compared to other floricane cultivars cultivated in the area [19].

The presented sensory study was carried out to establish a correlation between the chemical aroma profile of the tested varieties and the desirable characteristics of the fruit. The data that was collected from the sensory analysis was cross-referenced with the chemical analysis of volatile components of the fruit, which was carried out using multivariate analytic techniques. To generate new varieties that have an improved aromatic profile, breeders can use the results that were achieved as a guide while developing new varieties. The applied OPLS-HDA method has been shown to be very useful in a multiclass experiment, and it is also capable of being efficiently utilized in other types of research.

## 2. Materials and Methods

### 2.1. Sample Collection and Preparation

The six raspberry cultivars (Tula Magic, Glen Dee, San Rafael, Adelita, Cascade Harvest, Himbo Top), non-commercially grown in Serbia and most dominant in the system of integrated agricultural production, were harvested from a commercial plantation of the company Zeleni hit. Each row represented a single variety of berry. The distance between rows was 2.5 m, and the distance between plants in a row was 50 cm. The berry planting was carried out using a trellis system with integral protection, anti-hail nets, and a drip irrigation system. At the beginning of July 2021, berries were hand-harvested during the first year of vegetation. The berries were picked at the stage of technological maturity from different plants. Three fresh, randomly selected berries from each variety were used for sensory evaluation. The rest of the samples were stored at −18 °C until GC-MS analysis.

For each variety, 1 g of sample was homogenized and spiked with 1-chlorodecane (Sigma-Aldrich, Saint Louis, MO 63103, USA) solution in acetonitrile as an internal standard, at a concentration of 5 ppm. Then, 0.5 g of the homogenized sample was placed in a 2 mL headspace vial along with 0.25 g of NaCl (TUZLA, Bosna and Hercegovina). The vial was closed with a septum and incubated in a water bath at 39 °C for 1 h. The analysis was repeated for ten biological replicates (individual berries) for each variety. During incubation in the empty space of the vial, fiber emerged for the solid-phase microextraction with polydimethylsiloxane (PDMS) as an adsorbent. A manual SPME arrow injection kit was employed for incubation and GC-MS inlet injection of concentrated raspberry volatiles. After injection, the fiber was kept in the heated inlet for 20 s before starting the analysis for desorption, and for another four minutes after starting the analysis, to condition it for the next sample.

### 2.2. Solid-Phase Microextraction and Gas Chromatography–Mass Spectrometry Analysis

Extraction of volatiles from raspberry samples was carried out using divinylbenzene/carbon wide range/polydimethylsiloxane fiber with a thickness of 50/30 µm and a length of 10 mm (DVB/CAR/PDMS 50/30 μm). The fiber was conditioned before use according to the manufacturer’s manual. After injection, the fiber was kept in the heated inlet for 10 s before starting analysis for desorption and an additional four minutes after starting the analysis to condition it for the next sample. Blank samples were measured every day before analyzing the samples of raspberry varieties. The gas chromatography–mass spectrometry (GC-MS) experimental conditions were replicated from our previously published work [20]. GC–MS analysis was carried out using the Agilent 7890B GC system (Agilent Technologies, Santa Clara, CA, USA) equipped with a 5977 mass selective detector. Separation was achieved on a non-polar HP-5MSI capillary column (30 m × 0.25 mm, 0.25 μm film thickness). The oven was gradually heated from 60 °C to 240 °C at 3 °C per minute. As a carrier gas, helium was used. Inlet pressure was constant at 16.7 psi (flow 1.0 mL/min at 210 °C), and splitless mode was used. MS data were acquired in EI mode (70 eV at 230 °C) at an *m*/*z* range from 40 to 550 amu, the mass analyzer was at 150 °C, and the transfer line temperature was kept at 315 °C. The library search, mass spectral deconvolution, and extraction of detected compounds were conducted utilizing MSD ChemStation software, version E02.02 (Agilent Technologies, Santa Clara, CA, USA), NIST AMDIS software version 2.70, and the commercially available Adams04, NIST17, and Wiley07 libraries, which encompass approximately 500,000 spectra.

### 2.3. Sensory Characteristic Analysis

Sensory evaluation of fresh fruits from six raspberry cultivars was conducted using a standard nine-point hedonic scale [21]. A total of 55 panelists participated, each assessing the following attributes of every sample: appearance, color, aroma, taste, and overall impression. Sensory properties were rated according to the panelists’ individual perceptions using the following hedonic scale: 9—Like extremely; 8—Like very much; 7—Like moderately; 6—Like slightly; 5—Neither like nor dislike; 4—Dislike slightly; 3—Dislike moderately; 2—Dislike very much; 1—Dislike extremely.

### 2.4. Statistical Analysis

All analyses were conducted using SIMCA 18 (Sartorius, Germany) and Python 3.11 (Spyder 6 environment). To ensure panel reliability, a two-step screening was applied. First, principal component analysis (PCA) score plots were inspected to identify inconsistent assessors. A total of 55 raspberry consumers, highly educated volunteers from the Faculty of Chemistry, aged 25 to 83 (29 were men and 26 were women), agreed to participate in this study. All participants received detailed instructions on how to fill out the questionnaires before the evaluation, as well as three samples of each variety, a glass of water, and a waste container. A total of five judges were removed as global outliers across all attributes. Attribute-level outliers were subsequently removed using Tukey’s 1.5 × IQR rule within each variety–attribute combination [22].

Relationships among sensory attributes (appearance, color, flavor, taste, overall acceptability) were examined using Spearman’s rank correlation coefficient (ρ). Two complementary analyses were performed: judge-level correlations—each judge’s average rating across varieties was used to compute correlations, reflecting intra-judge consistency; variety-level correlations—mean attribute scores per variety (averaged across judges) were correlated, reflecting inter-attribute co-variation across products. The resulting 5 × 5 correlation matrices were visualized as heatmaps.

### 2.5. Multivariate Data Analysis

Feature extraction and alignment of GC–MS data were performed using MS-DIAL software version 5.5 [23]. For peak detection, a minimum peak height of 2500 was applied. Peaks were aligned across samples using a retention index tolerance of 25 and an electron ionization (EI) similarity tolerance of 70%. A 10-fold sample average/blank average change filter was implemented to exclude background features.

Associations between sensory perception and chemical composition were investigated using PCA, orthogonal partial least squares (OPLS) regression. Peak areas were normalized to the total ion current (TIC). After normalization, variables were mean-centered and Pareto-scaled; no log transformation was applied. Following MS-DIAL filtering, no missing values remained in the GC–MS matrix. The mean flavor ratings were used as Y-variables. Model performance was assessed using R^2^, Q^2^, cross-validation analysis of variance (CV-ANOVA), and permutation testing with 200 iterations. Models were considered statistically valid when CV-ANOVA yielded *p* < 0.05 and permuted R^2^ and Q^2^ values were lower than those of the original model.

Variable influence was interpreted through a loading plot with jack-knifed confidence intervals. To further refine discriminant features, a variable importance in projection (VIPpred) versus p (corr) plot was generated. Metabolites with VIPpred > 1 and |p (corr)| > 0.5 that also passed jack-knife confidence were considered key contributors to explaining variation in flavor ratings.

To examine GC–MS profile differences among the raspberry varieties, orthogonal partial least squares hierarchical discriminant analysis (OPLS-HDA) was performed following Forsgren et al. (2025) [24]. Traditional two-class models like orthogonal partial least squares discriminant analysis (OPLS-DA) struggle with multiclass problems, often requiring multiple manual comparisons. OPLS-HDA overcomes this problem by merging OPLS-DA with hierarchical clustering to create a decision tree, which allows for easy interpretation and analysis. It uses cross-validation to prevent overfitting and has shown strong performance against other methods, making it a powerful and user-friendly tool for analyzing multiclass data. This approach integrates OPLS-DA with recursive binary partitioning, using Cohen’s d distances of cross-validated predictions to build a dendrogram of interclass relationships. Each dendrogram branch corresponds to a binary OPLS-DA model, enabling hierarchical separation of varieties. For each split, both score and loading plots were evaluated to identify metabolites contributing to discrimination. Robustness was assessed with CV-ANOVA and permutation testing as described above. Discriminant variables were defined more stringently (VIPpred > 1.5, |p (corr)| > 0.6, and passing jack-knife confidence intervals).

## 3. Results and Discussion

### 3.1. Sensory Analysis

A sensory evaluation of six raspberry varieties—Adelita, Himbo Top, Tula Magic, Cascade Harvest, Glen Dee, and San Rafael—was conducted by 55 judges using a 9-point hedonic scale across five attributes: appearance, color, flavor, taste, and overall acceptability. Data were first screened for judge reliability using PCA, where five judges were identified as outliers and removed from all attributes. Attribute-level outliers were further excluded using Tukey’s 1.5 × IQR rule [22].

**Figure 1:**
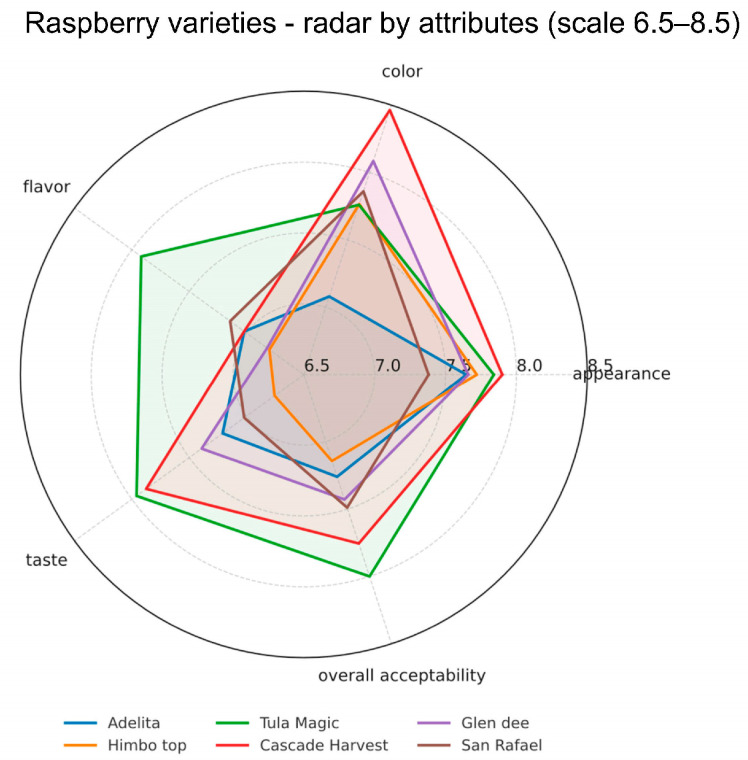
Radar plot showing mean sensory scores (9-point hedonic scale) for six raspberry varieties across five attributes.

The combined radar plot in Figure 1 shows the mean sensory scores. In addition to this visualization, Supplementary Table S1 reports the mean ± SD for each attribute × variety together with Tukey’s HSD (α = 0.05), and Supplementary Table S2 summarizes the corresponding one-way ANOVA results. Statistically significant variety effects were found for flavor, taste, and overall acceptability (ANOVA, *p* < 0.05), whereas appearance and color showed fewer pairwise separations. Tula Magic received the highest scores for flavor and also scored high for taste and overall acceptability, indicating that this variety was the most preferred in terms of eating quality. Cascade Harvest obtained the highest score for color, showing strong visual appeal, but had moderate scores for flavor and taste.

Adelita and Himbo Top had relatively uniform but lower scores across attributes, suggesting that they were less positively evaluated compared to the other varieties. San Rafael gave intermediate results, without a single attribute being dominant but with consistent acceptability across the panel. Glen Dee scored high for color but showed lower scores for flavor and taste, giving it a similar profile to Cascade Harvest but with slightly reduced overall acceptability.

Previous studies on raspberry sensory evaluation have established that key quality attributes driving consumer acceptance include sweetness, flavor intensity, and texture characteristics. Aaby et al. [25] characterized eight raspberry genotypes using descriptive sensory analysis and found that sour and green flavors versus chemical and cloying flavors described most of the sensory variation among genotypes. Their research demonstrated that titratable acidity correlated with acidic taste, astringency, and flavor intensity, while the soluble solids to titratable acidity ratio was positively correlated with sweet taste and negatively correlated with acidic taste and astringency. Similarly, Villamor et al. [26] identified that consumer liking of raspberries was primarily driven by high raspberry flavor, firmness, and sweetness, while disliking was related to high sour and aftertaste intensity. Research on raspberry cultivar comparisons has consistently shown significant varietal differences in sensory attributes. A study comparing five raspberry cultivars found substantial variations in sensory characteristics, with certain cultivars exhibiting superior flavor profiles and consumer acceptance [27].

These results show that raspberry quality is influenced by several sensory factors, consistent with the 00established literature demonstrating the complex interplay between chemical composition and sensory perception in berries. Visual attributes such as color and appearance do not always correspond to higher flavor or taste ratings, as confirmed by multiple studies showing that consumer preference is primarily driven by taste-related attributes rather than visual characteristics. Among the tested varieties, Tula Magic achieved the most favorable combination of sensory attributes, especially in flavor, which was closely linked to overall acceptability, reflecting patterns observed in previous raspberry quality studies where flavor intensity emerged as a key driver of consumer preference.

### 3.2. Correlation Analysis Between Sensory Attributes

To explore the relationships between sensory attributes, Spearman’s rank correlation coefficients (ρ) were computed. Two complementary perspectives were applied: judge-level correlations (consistency in how judges scored attributes) and variety-level correlations (relationships among variety means). Similar patterns have been reported in berry sensory evaluation studies, where trained panels consistently demonstrate strong correlations between taste-related attributes and overall acceptability [25,26].

**Figure 2:**
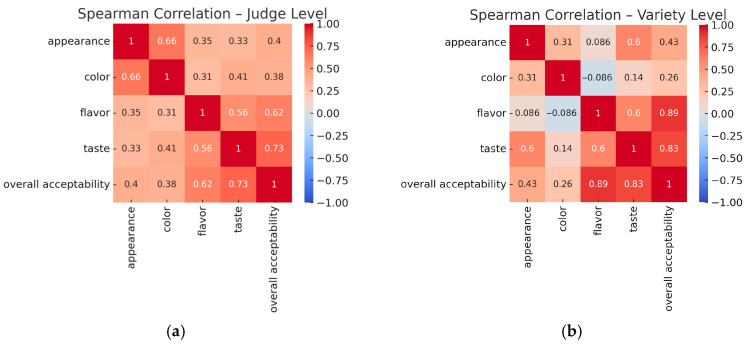
Spearman correlation heatmaps showing relationships among sensory attributes: (**a**) judge level (correlations across individual judges); (**b**) variety level (correlations across raspberry varieties).

The judge-level correlation matrix (Figure 2a) showed moderate-to-high positive correlations between most attributes. The strongest correlations were observed between taste and overall acceptability (ρ = 0.73) and between flavor and overall acceptability (ρ = 0.62). Appearance and color were also strongly correlated (ρ = 0.66), reflecting those judges who rated samples higher for color also tended to rate them higher for appearance. These relationships indicate that judges were generally consistent in linking hedonic impressions across multiple attributes.

At the variety level (Figure 2b), flavor showed a very strong positive correlation with overall acceptability (ρ = 0.89) and with taste (ρ = 0.60). Taste also correlated strongly with overall acceptability (ρ = 0.83). In contrast, color did not show a meaningful relationship with flavor (ρ = −0.09), and its correlation with overall acceptability was weaker (ρ = 0.26). These results suggest that flavor and taste were the main contributors to differences in overall acceptability across raspberry varieties, whereas color had less impact at the variety-mean level. This finding aligns with multiple studies on berry fruits demonstrating that consumer acceptance is primarily driven by taste and flavor attributes rather than visual characteristics [26,28,29].

Together, the two analyses provide complementary insights. Judge-level correlations demonstrate intra-panel consistency in how sensory impressions were linked, while variety-level correlations emphasize the relative importance of specific attributes for product differences. Both approaches confirm that flavor and taste were the strongest predictors of overall acceptability, whereas visual attributes (color and appearance) played a supporting but less decisive role. This hierarchical importance of sensory attributes has been consistently documented across multiple berry species and evaluation methods [26,28,29,30].

Variety-level trends were corroborated by pairwise scatterplots and leave-one-variety-out jack-knife analyses (Figure S1; Table S3), confirming that the positive associations of flavor and taste with overall acceptability were not driven by a single variety.

### 3.3. Multivariate Classification of Raspberry Varieties by SPME GC–MS Aroma Profiles

To evaluate whether raspberry varieties can be distinguished by their chemical composition, we analyzed their headspace volatile profiles obtained by SPME GC–MS (Table S4), a technique well suited for capturing aroma-active compounds.

**Figure 3:**
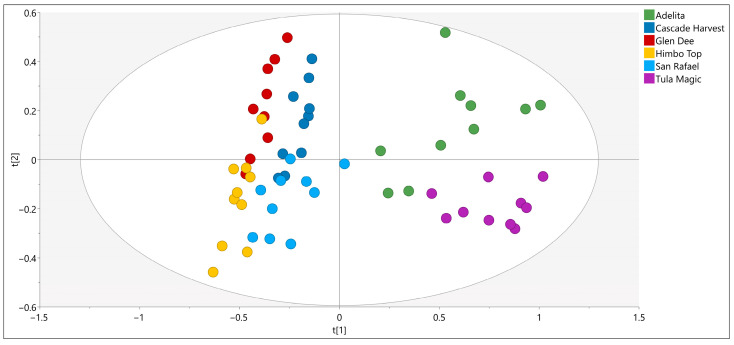
Principal component analysis (PCA) score plot of SPME GC–MS metabolite profiles from six raspberry varieties.

To explore the overall structure of this metabolite dataset, a PCA model with nine components was first examined, explaining 90% of the total variance. The PCA score plot (Figure 3) revealed no strong outliers and indicated only partial separation among raspberry varieties. While this suggested that the GC–MS profiles contained useful discriminatory information, unsupervised PCA alone did not provide sufficient resolution for reliable classification, highlighting the need for a supervised multivariate approach.

For this purpose, OPLS-HDA was applied. This method has been introduced only recently as an extension of OPLS-DA to handle multi-class problems in a hierarchical manner [24]. OPLS-HDA constructs pairwise OPLS-DA models and assembles them into a dendrogram, using distances derived from cross-validated predictions to represent how varieties cluster in chemical space. By structuring class separation as a series of binary comparisons, OPLS-HDA avoids the interpretability and prediction challenges often encountered in direct multi-class OPLS-DA models.

**Figure 4:**
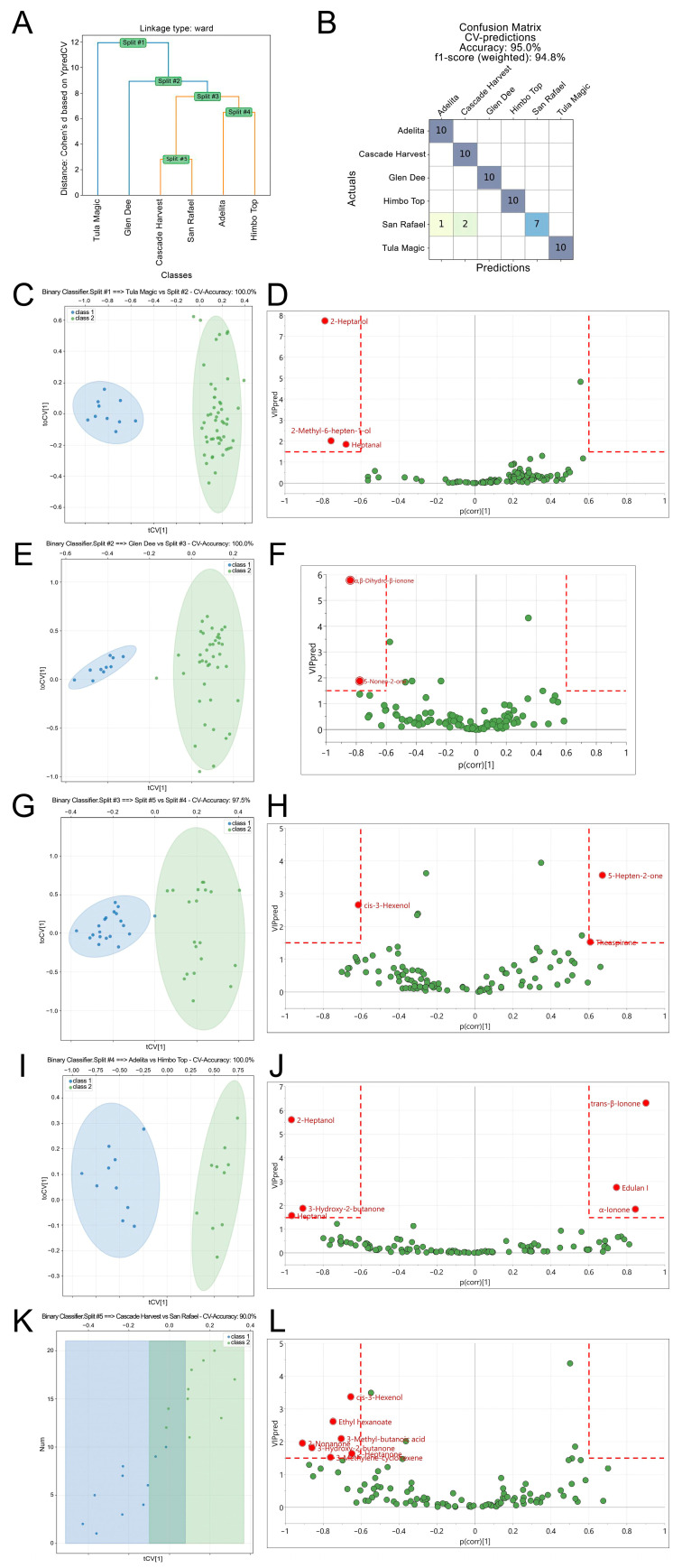
Hierarchical orthogonal partial least squares discriminant analysis (OPLS-HDA) of SPME GC–MS metabolite profiles from six raspberry varieties. (**A**) OPLS-HDA dendrogram showing five principal binary splits based on cross-validated model distances. (**B**) Confusion matrix summarizing cross-validation performance. (**C**,**E**,**G**,**I**,**K**) Cross-validated OPLS-DA (CV-score) plots from individual binary classifiers at each split, showing consistent pairwise separation between varieties. (**D**,**F**,**H**,**J**,**L**) VIPpred vs. p (corr) plots for the corresponding binary models, highlighting discriminating metabolites.

The dendrogram (Figure 4A) revealed five principal binary splits. Each split was represented by a two-class OPLS-DA model that displayed clear separation in cv-score plots (Figure 4C–L, left panels). Metabolites contributing to each separation were identified in VIPpred vs. p (corr) plots (Figure 4C–L, right panels). Discriminating variables were selected according to strict criteria: VIPpred > 1.5, |p (corr)| > 0.6, and jack-knife confidence intervals not crossing zero. These thresholds ensured that only features both predictive and stable under cross-validation were retained for interpretation.

In metabolomics studies, volcano plots are frequently used to identify discriminant features by plotting fold-change against *p*-values, enabling rapid visual detection of metabolites that vary significantly between groups [31]. However, this univariate strategy can produce false discoveries and is highly sensitive to multiple testing corrections. By contrast, the OPLS-based criteria—VIPpred, p (corr), and jack-knife stability—are derived directly from the supervised model, ensuring consistency with the multivariate discrimination task [32]. The use of jack-knife intervals further confirms that the retained variables are statistically stable, a feature lacking in volcano-based selection.

Key discriminating metabolites identified by OPLS-HDA splits in SPME GC–MS chromatograms of raspberry varieties (Table 1).

Table 1 summarizes the metabolites most strongly contributing to each binary split in the OPLS-HDA dendrogram. These discriminating features provide a chemically interpretable explanation of how raspberry varieties were classified.

In Split #1, Tula Magic was distinguished by elevated levels of 2-heptanol, heptanal, and 2-methyl-6-hepten-1-ol. According to odor databases, 2-heptanol is described as fresh, lemongrass-like, herbal, floral, fruity, and green, while heptanal has been characterized as fresh, aldehydic, fatty, green, herbal, and ozone-like [33]. In addition, 2-methyl-6-hepten-1-ol is reported to exhibit oily-green, herbaceous-citrusy notes resembling coriander and unripe berries [34]. Combined, these volatiles suggest a contribution to fresh green and fruity aroma qualities in Tula Magic.

In Split #2, Glen Dee was rich in α,β-dihydro-β-ionone and 5-nonen-2-one. According to odor databases, dihydro-β-ionone is described as earthy, woody, mahogany/orris, and dry-amber [34], while 5-nonen-2-one has been associated with fruity and cheesy notes in raspberries [35].

In Split #3, the Adelita + Himbo Top branch was characterized by elevated (Z)-3-hexenol, a green-leaf volatile associated with grassy notes, whereas 6-methyl-5-hepten-2-one (fatty, green, citrus) and theaspirane (tea-like, herbal, green) [33] differentiated the Cascade Harvest + San Rafael branch.

In Split #4, Adelita was characterized by higher levels of lipid-derived volatiles—2-heptanol (fresh, lemongrass-like, herbal, floral, fruity, green), 3-hydroxy-2-butanone (buttery), and heptanal (fresh, aldehydic, fatty, green, herbal, ozone-like)—whereas Himbo Top was rich in trans-β-ionone (violet sweet floral), edulan I (floral, rose), and α-ionone (sweet-woody, floral, violet/orris, tropical-fruity) [33].

In Split #5, Cascade Harvest was characterized by a broader volatile profile, including (Z)-3-hexenol (intense grassy–green odor), ethyl hexanoate (sweet, fruity, pineapple, waxy, green, banana), 3-methylbutanoic acid (unpleasant, sweaty/foot-like), 3-methylene-cyclohexene, 3-hydroxy-2-butanone (buttery), 2-nonanone (fresh, sweet, green, weedy, earthy, herbal), and 2-heptanone (fruity, spicy, sweet, herbal, coconut, woody) [33], suggesting a more complex aroma composition.

Overall, the hierarchical distribution of rich metabolites mirrors their biosynthetic origins: lipid-derived aldehydes and alcohols were key for early splits, while apocarotenoids explained finer varietal distinctions. This supports the biological interpretability of the OPLS-HDA classification and confirms that the selection strategy captured both statistically robust and aroma-relevant markers. Consequently, the combination of SPME GC–MS profiling with the recently introduced OPLS-HDA framework provided a rigorous and interpretable approach for distinguishing raspberry varieties and identifying reliable discriminating metabolites.

### 3.4. Correlation of GC–MS Aroma Profiles with Sensory Evaluation by OPLS

**Figure 5:**
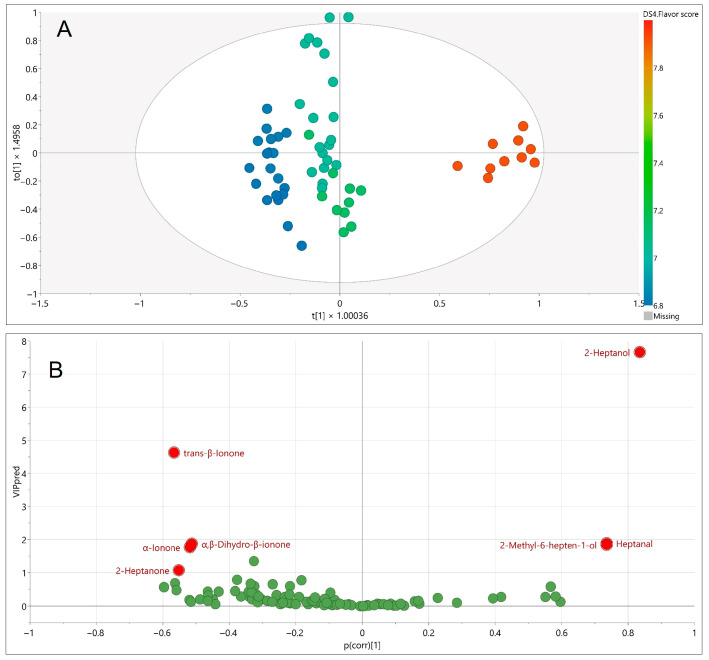
Orthogonal partial least squares (OPLS) regression model linking SPME GC–MS volatile metabolite profiles to mean sensory flavor scores of raspberry cultivars. (**A**) OPLS score plot (**B**) VIPpred vs. p (corr) plot, highlighting discriminating volatiles most strongly associated with flavor perception.

To directly connect chemical composition with sensory perception, an orthogonal projection to latent structures (OPLS) regression model was constructed using the mean flavor scores from the trained judge panel as the response variable and SPME GC–MS metabolites as predictors (Figure 5). The model consisted of one predictive and two orthogonal components, with excellent explanatory and predictive performance (R^2^Y = 0.965, Q^2^ = 0.950). Model validity was confirmed by CV-ANOVA (F = 166.7, *p* = 1.4 × 10^−32^) and by permutation testing, which excluded overfitting.

Volatile metabolites most strongly linked to sensory flavor scores (Table 2) were identified using VIPpred, p (corr), and jack-knife stability. Three compounds emerged as positive contributors: 2-heptanol (fresh, lemongrass-like, herbal, floral, fruity, green), 2-methyl-6-hepten-1-ol (oily-green, herbaceous, citrusy), and heptanal (fresh, aldehydic, fatty, green, herbal). These volatiles are products of lipid oxidation and have been previously reported in raspberry aroma profiles [6]. Their sensory qualities suggest a contribution to freshness and complexity, consistent with the positive alignment observed with panel scores.

Conversely, several compounds showed negative contributions, including trans-β-ionone (violet, sweet, floral), α-ionone (woody, floral), α,β-dihydro-β-ionone (earthy, woody), and 2-heptanone (fruity, spicy, sweet, herbal, coconut, woody). These apocarotenoids and ketones are well established as raspberry volatiles [6], and the ionones in particular are regarded as key impact odorants contributing violet–floral and raspberry-like nuances. However, in the present model, higher relative levels of these compounds were associated with slightly lower sensory scores. This finding does not imply that their presence reduces flavor quality; rather, it indicates that excessive concentrations may disrupt sensory balance, while moderate levels enhance overall appeal.

Importantly, all raspberry cultivars in this study received high flavor scores, emphasizing that a negative correlation in the OPLS model does not equate to inferior aroma. Instead, the results highlighted that raspberry flavor quality is determined by a delicate balance between positively and negatively contributing volatiles, in line with previous studies reporting complex interactions among aroma-active compounds [6,36].

## 4. Conclusions

According to the results of 55 volunteer judges in sensory evaluations of six raspberry varieties—Adelita, Himbo Top, Tula Magic, Cascade Harvest, Glen Dee, and San Rafael—Tula Magic had the highest overall score, especially when considering aroma, taste, and overall acceptability. However, if we used color alone as a parameter, it is, next to Himbo Top, the lowest-rated. The presented results indicate that flavor and taste were the primary factors influencing overall acceptability among raspberry varieties, while color had a diminished effect at the variety-mean level.

The newly introduced method of orthogonal projections to latent structures–hierarchical discriminant analysis (OPLS-HDA) was applied to multivariate classification of raspberry varieties via SPME GC–MS. Dealing with multi-class problems in a hierarchical manner, this method successfully built a large number of OPLS-DA models to compare each class with each other and, based on that, determine their similarities and, ultimately, the components of differentiation. Each split in the dendrogram was presented with a separate OPLS-DA model, which allowed us to identify biomarkers from VIPpred vs. p (corr) plots. All models were validated using CV-ANOVA and a permutation test.

In OPLS, the results of the SPME GC–MS volatile metabolite profiles were linked to the mean sensory flavor scores of the raspberry cultivars. 2-heptanol, 2-methyl-6-hepten-1-ol, and heptanal were attributed positive sensory flavor scores, while terpene components, characteristic of raspberry and fruit flavors, were assigned negative ones. All raspberry cultivars in this study received high flavor scores, indicating that a negative correlation in the OPLS model does not imply an inferior aroma. The findings indicate that raspberry flavor quality is influenced by a delicate balance of positively and negatively contributing volatiles, consistent with previous studies that report complex interactions among aroma-active compounds.

## Figures and Tables

**Table 1:** OPLS-HDA discriminating metabolites by hierarchical split with effect direction, VIPpred, p (corr), and jack-knife CI status.

Split	Binary Comparison	Metabolite (Enriched in …)	Odor Description	VIPpred	p (Corr)	JK CI
#1	Tula Magic vs. All others	2-Heptanol (enriched in Tula Magic)	Fresh, lemongrass-like, herbal, floral, fruity, green	7.73	−0.79	Pass
#1	Tula Magic vs. All others	Heptanal (enriched in Tula Magic)	Fresh, aldehydic, fatty, green, herbal, ozone-like	1.85	−0.68	Pass
#1	Tula Magic vs. All others	2-Methyl-6-hepten-1-ol (enriched in Tula Magic)	Oily-green, herbaceous, citrus-like	2.02	−0.76	Pass
#2	Glen Dee vs. (Split #3 branch)	α,β-Dihydro-β-ionone (enriched in Glen Dee)	Earthy, woody/orris-like	5.78	−0.84	Pass
#2	Glen Dee vs. (Split #3 branch)	5-Nonen-2-one (enriched in Glen Dee)	Fruity, cheesy	1.88	−0.78	Pass
#3	(Split #4 branch) vs. (Split #5 branch)	(Z)-3-Hexenol (enriched in Split #4: Adelita + Himbo Top)	Fresh green, leafy, grassy	2.66	−0.62	Pass
#3	(Split #4 branch) vs. (Split #5 branch)	6-Methyl-5-hepten-2-one (enriched in Split #5: Cascade Harvest + San Rafael)	Fatty, green, citrus-like	3.57	0.67	Pass
#3	(Split #4 branch) vs. (Split #5 branch)	Theaspirane (enriched in Split #5: Cascade Harvest + San Rafael)	Tea-like, herbal, green, woody/spicy	1.53	0.61	Pass
#4	Adelita vs. Himbo Top	2-Heptanol (enriched in Adelita)	Fresh, lemongrass-like, herbal, floral, fruity, green	5.60	−0.97	Pass
#4	Adelita vs. Himbo Top	3-Hydroxy-2-butanone (enriched in Adelita)	Buttery, creamy	1.88	−0.91	Pass
#4	Adelita vs. Himbo Top	Heptanal (enriched in Adelita)	Fresh, aldehydic, fatty, green, herbal, ozone-like	1.58	−0.96	Pass
#4	Adelita vs. Himbo Top	trans-β-Ionone (enriched in Himbo Top)	Violet, sweet, floral/raspberry-like	6.31	0.90	Pass
#4	Adelita vs. Himbo Top	Edulan I (enriched in Himbo Top)	Floral, rose-like	2.76	0.75	Pass
#4	Adelita vs. Himbo Top	α-Ionone (enriched in Himbo Top)	Woody, floral/raspberry-like	1.84	0.84	Pass
#5	Cascade Harvest vs. San Rafael	(Z)-3-Hexenol (enriched in Cascade Harvest)	Fresh green, leafy, grassy	3.37	−0.66	Pass
#5	Cascade Harvest vs. San Rafael	Ethyl hexanoate (enriched in Cascade Harvest)	Fruity, apple/pineapple, sweet	2.61	−0.75	Pass
#5	Cascade Harvest vs. San Rafael	3-Methylbutanoic acid (enriched in Cascade Harvest)	Pungent, sweaty, cheesy	2.10	−0.71	Pass
#5	Cascade Harvest vs. San Rafael	3-Methylene-cyclohexene (enriched in Cascade Harvest)	Terpenic/hydrocarbon	1.53	−0.76	Pass
#5	Cascade Harvest vs. San Rafael	3-Hydroxy-2-butanone (enriched in Cascade Harvest)	Buttery, creamy	1.81	−0.86	Pass
#5	Cascade Harvest vs. San Rafael	2-Nonanone (enriched in Cascade Harvest)	Fresh, sweet, green, weedy, earthy, herbal	1.95	−0.91	Pass
#5	Cascade Harvest vs. San Rafael	2-Heptanone (enriched in Cascade Harvest)	Fruity, spicy, sweet, herbal, coconut, woody	1.64	−0.65	Pass

Notes: VIPpred = variable importance on the predictive component; p (corr) = correlation-scaled loading (sign indicates direction; positive corresponds to the class listed in parentheses as ‘enriched in’); JK CI = jack-knife 95% confidence interval for the predictive coefficient (Pass = CI excludes zero).

**Table 2:** Volatile metabolites most strongly contributing to sensory flavor score prediction by OPLS regression.

Compound	Class	Aroma Descriptor(s)	Contribution to Flavor Score
2-Heptanol	Alcohol	Fresh, lemongrass-like, herbal, floral, fruity, green	Strong Positive
2-Methyl-6-hepten-1-ol	Alcohol	Oily-green, herbaceous-citrusy notes	Positive
Heptanal	Aldehyde	Fresh, aldehydic, fatty, green, herbal	Positive
trans-β-Ionone	Apocarotenoid	Violet, sweet, floral	Negative
α-Ionone	Apocarotenoid	Woody, floral	Negative
α,β-Dihydro-β-ionone	Apocarotenoid	Earthy, woody	Negative
2-Heptanone	Ketone	Fruity, spicy, sweet, herbal, coconut, woody	Negative

## Data Availability

The original contributions presented in this study are included in the article/Supplementary Materials. Further inquiries can be directed to the corresponding author.

## Supplementary Materials

The following supporting information can be downloaded at: https://www.mdpi.com/article/10.3390/foods14213599/s1, Table S1: Mean ± SD sensory scores by attribute and variety; Table S2: One-way ANOVA by attribute; Table S3: Spearman and Kendall correlation coefficients between variety-level sensory means with jack-knife and bootstrap robustness estimates; Table S4: Volatile profiles of six raspberry cultivars obtained through SPME GC–MS. Figure S1. Pairwise scatterplots of variety-level sensory means for five attributes (appearance, color, flavor, taste, and overall acceptability).

## Abbreviations

The following abbreviations are used in this manuscript:

SPME	Solid-phase microextraction
HS-SPME	Head Space SPME
GC	Gas chromatography
GC–MS	Gas Chromatography–Mass Spectrometry
PCA	Principal component analysis
OPLS	Orthogonal partial least squares
CV-ANOVA	Cross-validation analysis of variance
VIPpred	Variable importance in projection
OPLS-HDA	Orthogonal partial least squares hierarchical discriminant analysis
OPLS-DA	Orthogonal partial least squares discriminant analysis
