# Supplementary material for: Aroma Profiling and Sensory Association of Six Raspberry Cultivars Using HS-SPME/GC-MS and OPLS-HDA
Source: Foods. 2025 Oct 22;14(21):3599. doi: 10.3390/foods14213599 (PMC12608649; doi:10.3390/foods14213599)
Supplement: Supplementary file 1 [file foods-14-03599-s001.zip › Tables S1-3; Figure S1.pdf]

Aroma Profiling and Sensory Association of Six Raspberry Cultivars Using HS-SPME/GC-MS and OPLS-HDA

Supplementary Materials

Table S1. Mean ± SD sensory scores by attribute and variety.

Attribute	Variety	Mean ± SD
appearance	Adelita	7.65 ± 1.28[A]
appearance	Cascade Harvest	7.90 ± 1.42[A]
appearance	Glen dee	7.66 ± 1.24[A]
appearance	Himbo top	7.72 ± 1.26[A]
appearance	San Rafael	7.38 ± 1.60[A]
appearance	Tula Magic	7.84 ± 1.04[A]
color	Adelita	7.08 ± 1.51[A]
color	Cascade Harvest	8.46 ± 0.64[B]
color	Glen dee	8.09 ± 0.69[B]
color	Himbo top	7.77 ± 0.81[C]
color	San Rafael	7.86 ± 1.01[B]
color	Tula Magic	7.76 ± 1.12[C]
flavor	Adelita	7.02 ± 1.83[A]
flavor	Cascade Harvest	7.02 ± 1.74[A]
flavor	Glen dee	6.82 ± 1.34[A]
flavor	Himbo top	6.80 ± 1.54[A]
flavor	San Rafael	7.14 ± 1.46[A]
flavor	Tula Magic	7.92 ± 1.06[B]
overall acceptability	Adelita	7.26 ± 1.35[A]

overall acceptability	Cascade Harvest	7.76 ± 1.22^{A}
overall acceptability	Glen dee	7.43 ± 0.91^{A}
overall acceptability	Himbo top	7.14 ± 1.22^{A}
overall acceptability	San Rafael	7.49 ± 0.94^{A}
overall acceptability	Tula Magic	8.00 ± 0.00^{B}
taste	Adelita	7.21 ± 1.62^{A}
taste	Cascade Harvest	7.88 ± 1.41^{A}
taste	Glen dee	7.39 ± 0.93^{A}
taste	Himbo top	6.76 ± 1.57^{B}
taste	San Rafael	7.02 ± 1.57^{B}
taste	Tula Magic	7.96 ± 1.14^{A}

Note: Values are mean ± standard deviation. Different superscript letters within an attribute indicate significant differences among varieties (Tukey's HSD, $\alpha = 0.05$).

Table S2. One-way ANOVA by attribute.

Attribute	df_between	df_within	F	p
appearance	5	292	0.958	4.441e-01
color	5	279	9.209	3.972e-08
flavor	5	291	3.642	3.273e-03
taste	5	283	5.602	6.103e-05
overall acceptability	5	263	3.223	7.655e-03

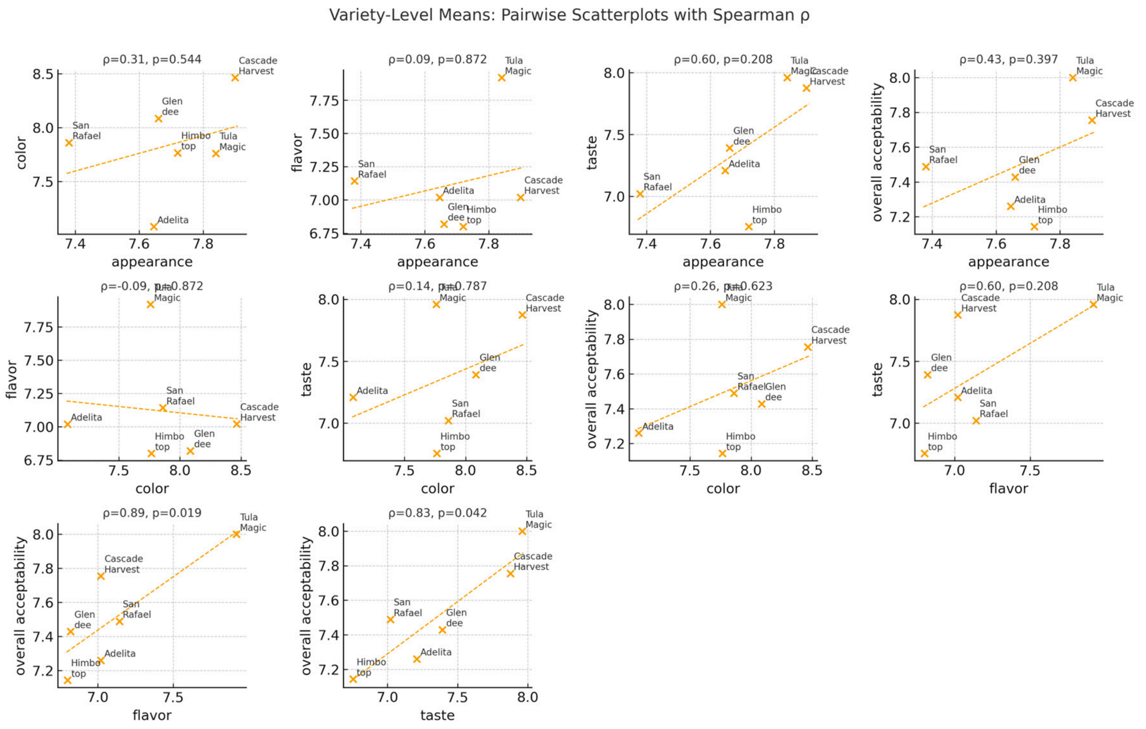

Figure S1. **Pairwise scatterplots of variety-level sensory means for five attributes (appearance, color, flavor, taste, and overall acceptability).**
Each point represents one raspberry variety (n = 6). Panel titles show Spearman's rank correlation coefficient (ρ) and p-value. Dashed lines indicate least-squares fits. The plots illustrate the stability of the positive relationships—particularly between flavor, taste, and overall acceptability—verified by jackknife and bootstrap resampling (see Table S3).

Table S3. Spearman and Kendall correlation coefficients between variety-level sensory means with jackknife and bootstrap robustness estimates.
For each attribute pair, the table lists Spearman's ρ (p-value), Kendall's τ (p-value), jackknife ρ range (leave-one-variety-out), and 95 % bootstrap confidence interval of ρ (1000 resamples, n = 6).

Attribute X	Attribute Y	Spearman ρ	Spearman p	Kendall τ	Kendall p	Jackknife ρ min	Jackknife ρ max	Bootstrap 95% CI low	Bootstrap 95% CI high	n varieties
appearance	color	0.314	0.5441	0.2	0.7194	-0.2	0.6	-1	1	6
appearance	flavor	0.086	0.8717	-0.067	1	-0.4	0.5	-1	0.8	6
appearance	taste	0.6	0.208	0.467	0.2722	0.4	0.9	-0.6	1	6
appearance	overall acceptability	0.429	0.3965	0.2	0.7194	0.1	0.6	-1	1	6

color	flavor	-0.086	0.8717	-0.067	1	-0.3	0.3	-1	0.92	6
color	taste	0.143	0.7872	0.2	0.7194	-0.1	0.7	-0.8	1	6
color	overall acceptability	0.257	0.6228	0.2	0.7194	-0.1	0.8	-0.8	1	6
flavor	taste	0.6	0.208	0.467	0.2722	0.3	0.9	-0.636	1	6
flavor	overall acceptability	0.886	0.0188	0.733	0.0556	0.8	0.9	0.2	1	6
taste	overall acceptability	0.829	0.0416	0.733	0.0556	0.7	1	0.032	1	6
